# Editorial: Technology advancements, social media and innovations in uro-oncology and endourology

**DOI:** 10.3389/fsurg.2022.1069746

**Published:** 2022-11-07

**Authors:** B M Zeeshan Hameed, Nithesh Naik, Bhavan Prasad Rai, Bhaskar K. Somani

**Affiliations:** ^1^ Department of Urology, Father Muller Medical College, Mangalore, Karnataka, India; ^2^ iTRUE (International Training and Research in Uro-Oncology and Endourology) Group, Manipal, India; ^3^ Curiouz TechLab Private Limited, BIRAC-BioNEST, Manipal Government of Karnataka Bioincubator, Manipal, India; ^4^ Department of Mechanical and Industrial Engineering, Manipal Institute of Technology, Manipal Academy of Higher Education, Manipal, India; ^5^ Department of Urology, Freeman Hospital, Newcastle Upon Tyne, United Kingdom; ^6^ Department of Urology, University Hospital Southampton NHS Trust, Southampton, United Kingdom

**Keywords:** kidney calculi, ureteroscopy, PCNL, AI, social media, BPH, telemedicine, prostate cancer

**Editorial on the Research Topic** Technology advancements, social media and innovations in uro-oncology and endourology By Hameed BMZ, Naik N, Rai BP, Somani BK. (2022) Front. Surg. 9: 1069746. doi: 10.3389/fsurg.2022.1069746

Urology is making rapid technological advances with innovations in the field of uro-oncology and endourology. This is amply supported by social media in creating awareness of these new advancements. As the landscape of minimally invasive urology changes, to highlight and address this area of research, this Research Topic in “Frontiers in Surgery” was dedicated to collecting high-quality scientific contributions focusing mainly on technology advancements, social media, and innovations in Uro-oncology and Endourology.

The research topic includes articles (Gan et al. 2022, Cao et al. 2022, Wu et al. 2022, Chaudhary et al. 2022, Naik et al. 2022, Naik et al. 2022, Hameed et al. 2022, Hameed et al. 2022, Durutovic et al. 2022, Sparwasser et al. 2022, Zhou et al. 2021, Hu et al. 2022, Mao et al. 2022, Deng et al. 2022) that highlight three papers on endoscopic management, two of which are related to benign prostate hyperplasia (BPH) (Gan et al. 2022, Cao et al. 2022, Wu et al. 2022). A single-center experience on the role of “Immediate Transurethral Plasma Kinetic Enucleation of the Prostate Gland (i-TUPKEP) for Treatment of Benign Prostatic Hyperplasia-Associated Massive Hemorrhage (BHM)” Gan et al. (2022) carried a retrospective analysis of 49 patients. The preliminary data suggest that i-TUPKEP is a feasible technique for BHM and relieving BPH symptoms. Cao et al. (2022) in their study identified the role of “Transurethral Incision of the Bladder Neck (TUIBN) at Three Points with a Needle-Type Electrode for Bladder Neck Contracture (BNC)” Cao et al. (2022). 53 patients showed successful treatment using TUIBN, and the study concludes that it is a safe and reliable option in patients with BNC. Wu et al. (2022) in their endoscopic study focused on the “Removal of large fibrotic bladder blood clots using prostatic tissue morcellator under real-time ultrasound guidance”. Real-time ultrasound guidance combined with prostate tissue morcellator shows it to be the safe and effective procedure for the removal of large fibrotic bladder clots in all nine patients.

The research topic includes six articles that highlight the role of innovative technologies in urological patient care (Chaudhary et al. 2022, Naik et al. 2022, Naik et al. 2022, Hameed et al. 2022, Hameed et al. 2022, Durutovic et al. 2022, Sparwasser et al. 2022). Chaudhary et al. 2022 look at YouTube videos as a source of patient information for ureteric stent placement Chaudhary et al. (2022). The observations of their study concluded that the majority of videos are of poor overall quality and lack pertinent information suggesting the need for creating comprehensive unbiased videos. Naik et al. (2022) in the review raise an important concern by considering the various aspect of artificial intelligence (AI) on the ownership of responsibility and legal and ethical considerations in healthcare Naik et al. (2022). Naik et al. (2022) highlighted the patient's perception and feedback on the role of telemedicine and telehealth Naik et al. (2022). They examined recent research on video consulting in urology clinics for hematuria referrals and follow-up appointments for benign prostatic hyperplasia (BPH), kidney stone disease (KSD), and urinary tract infections (UTIs) and found that they are extremely acceptable and satisfactory. Telemedicine, a competent, cost-effective patient-care technology, has been effectively applied in numerous healthcare settings and specialties for such patients Naik et al. (2022).

Role of 3D printing in endourology is discussed as being helpful to visualize patient anatomy and aiding in pre-operative planning and training Hameed et al. (2022). The application of virtual reality augmented reality and mixed reality in endourology and urolithiasis is also discussed Hameed et al. (2022). Simulation-based training with these new immersive technologies allows improved training and should be a part of the curriculum. Otas et al. (2022) discussed 3D imaging segmentation and 3D rendering process for precise PCNL puncture, which may have widespread use and adoption for complex stone treatment Durutovic et al. (2022). The assessment of a novel smart-glass-based point-of-care fusion approach for mixed reality-assisted targeted prostate biopsy, suggests that this has the potential to improve accuracy for the detection of prostate cancer Sparwasser et al. (2022).

The research by Zhou et al. (2021) evaluates and predicts cancer-specific survival among patients with radical prostatectomy for prostate cancer. Nearly 96,000 patients were considered in the study and were divided into training and validation cohorts, to develop a competing risk nomogram to predict cancer-specific death for these patients Zhou et al. (2021). Hu et al. (2022) performed a retrospective analysis of 213 patients who underwent balloon dilatation for ureteral stricture and show that the long-term effect of three stents was better than that of single or double stents Hu et al. (2022). A successful robotic-assisted modified Y-shaped ileal orthotopic neobladder reconstruction is presented in 21 patients by Mao and colleagues Mao et al. (2022) A case report of renal pseudoaneurysm after flexible ureteroscopy and lasertripsy is mentioned by Deng et al. They suggest reducing operative time and intrarenal pressure to prevent these complications Deng et al. (2022).

Through a string of articles focussing on technology, innovation, and social media this special issue addresses a series of topics in uro-oncology and endourology. These outcomes will be useful for urology trainees and consultants alike and are likely to help and enhance their clinical practice. It will help them gain insight into newer and cutting-edge advances in the field of minimally invasive urology.

